# Assessing Referrals to a Trauma and Orthopaedic Department: Evaluation of a Traffic Light System for Virtual Fracture Clinic in the Emergency Department and Urgent Care

**DOI:** 10.7759/cureus.41316

**Published:** 2023-07-03

**Authors:** Effie Menyah, Sean M Garcia, Ann McCormack, Babajide Taiwo, Mohamed Aly, Walid Kamel, Baljinder S Dhinsa

**Affiliations:** 1 Trauma and Orthopaedics, William Harvey Hospital, Ashford, GBR; 2 Orthopaedics, Alexandria University, Alexandria, EGY

**Keywords:** emergency medicine and trauma, orthopaedic referrals, urgent treatment center, minor injury unit, virtual fracture clinic

## Abstract

Background

The Trauma and Orthopaedic (T&O) on-call service receives referrals from the emergency department (ED), general practice (GP) and urgent treatment centres (UTCs) and requests for inpatient reviews. The virtual fracture clinic (VFC) pathway allows ED and UTC clinicians to assess, discharge and refer when necessary. For VFC, the on-call orthopaedic consultant reviews the cases the next working day and makes an appropriate plan. This pathway consists of a traffic light system, in which practitioners can either safely discharge with written advice (green), refer to the VFC (yellow) or refer to the on-call team (red).

Method

The aim of this study was to assess how the VFC pathway was being utilised. All referrals to the T&O on-call team over three weeks were evaluated retrospectively. The following referrals were excluded: fractured femur, head injury, trauma calls and back pain pathway. The following data were collected: patient details, diagnosis, referral source, reason for referral, plan, double booking with VFC and appropriateness.

Results

A total of 191 referrals were analysed. Most referrals are from the ED (51%) and UTC (23%). Of the referrals, 39% were deemed to be inappropriate. Of the inappropriate referrals, 35% should have been referred directly to the VFC rather than the on-call team. A significant minority (7%) of inappropriate referrals were referred to the on-call team and VFC.

Conclusion

Education and collaboration are required with the ED and UTC to ensure the proper use of the VFC pathway. Immediate radiograph reporting may also be beneficial.

## Introduction

Musculoskeletal complaints are common presentations to the emergency department (ED). They range from low-severity strains to high-severity trauma [1,2]. High volumes of presentations to the ED are known to result in overcrowding and delays to care [3,4]. The utilisation of urgent treatment centre (UTC) services, specifically minor injuries units (MIUs), can reduce this burden [5] by managing and discharging, where appropriate, less severe musculoskeletal complaints.

At our institution, the Trauma and Orthopaedic (T&O) on-call service receives referrals from the ED, general practice (GP) and urgent treatment centres (UTCs) and requests for inpatient reviews. Patients seen in the UTC and ED with musculoskeletal complaints have their management streamlined via a virtual fracture clinic (VFC) emergency management pathway. Virtual fracture clinics are well established in the United Kingdom with multiple studies confirming reasonable patient satisfaction [6,7], good clinical outcomes, overall improved efficiency of trauma services, cost savings [6,8], safety [9], reduced unplanned ED re-attendances [10] and invaluable time for the T&O service to focus on more complex cases [11].

Our Trust's VFC pathway consists of a traffic light system of musculoskeletal diagnoses, from which practitioners can safely discharge with written advice (green), refer to the VFC (yellow) or refer to the on-call team (red) if warranted. Our current pathway version is the latest iteration that includes more diagnoses compared to previous iterations. The virtual fracture clinic pathway allows ED and UTC clinicians to assess, manage and discharge or refer to the T&O on-call service when necessary. For VFC, the healthcare practitioner reviewing the patient is responsible for making the referral electronically using the Trust's electronic medical records (EMRs). The practitioner provides their history, examination findings and management plan performed. The on-call orthopaedic consultant will review the cases with X-rays the next working day and make an appropriate plan in keeping with the British Orthopaedic Association (BOA) Standards for Trauma for fracture clinic services [12]. Should a patient in VFC require an early investigation or intervention such as a CT scan or surgery, this will be arranged through VFC, and a face-to-face appointment is made if necessary. Patients are informed via telephone and a letter in the post with the outcome, and this is added to their EMR for clinical documentation purposes. Patients reviewed by the on-call team/consultant are never referred to VFC as this will duplicate or result in differing consultant decisions and plans. Face-to-face fracture clinic appointments are made for such patients if necessary.

An example of a typical patient's pathway could be as follows. A patient comes into the ED with a displaced distal radius fracture. The patient is triaged and seen by the ED where fracture reduction is attempted and put into a back slab with repeat X-rays as per the yellow pathway. The patient is referred to VFC and reviewed by the on-call orthopaedic consultant virtually the following day. On review, the consultant notes this is an intra-articular volar displaced fracture and decides on surgical management. The patient is added to the trauma board and informed of the plan.

Our study aimed to evaluate the appropriateness of referrals received by the T&O on-call team in relation to the VFC Matrix and determine if the pathway was being used correctly.

## Materials and methods

All referrals to the T&O on-call team over three weeks were assessed retrospectively. This included referrals from the ED, UTC, general practitioners and inpatient wards. To assess the use of the VFC Matrix, data was collected from the departmental Trauma Drive, a system used to record each referral to the T&O team and manage trauma theatre lists.

**Figure 1:**
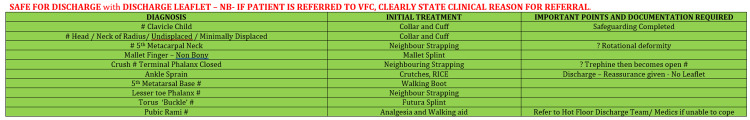
Virtual Fracture Clinic Emergency Fracture Management Matrix: Green Pathway Adapted from East Kent Hospitals University Foundation Trust Virtual Fracture Clinic Matrix for management of specific fractures, with permission. Designed by Jenni Finlay (Trauma Nurse Practitioner, Trauma and Orthopaedics Department, William Harvey Hospital, East Kent Hospitals University Foundation Trust).

**Figure 2:**
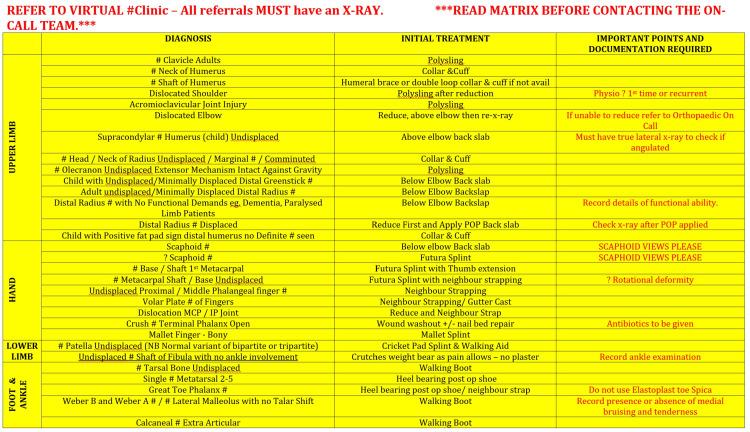
Virtual Fracture Clinic Emergency Fracture Management Matrix: Yellow Pathway Adapted from East Kent Hospitals University Foundation Trust Virtual Fracture Clinic Matrix for management of specific fractures, with permission. Designed by Jenni Finlay (Trauma Nurse Practitioner, Trauma and Orthopaedics Department, William Harvey Hospital, East Kent Hospitals University Foundation Trust).

All referrals during this period from the green and yellow pathways were included (Figures 1, 2).

**Figure 3:**
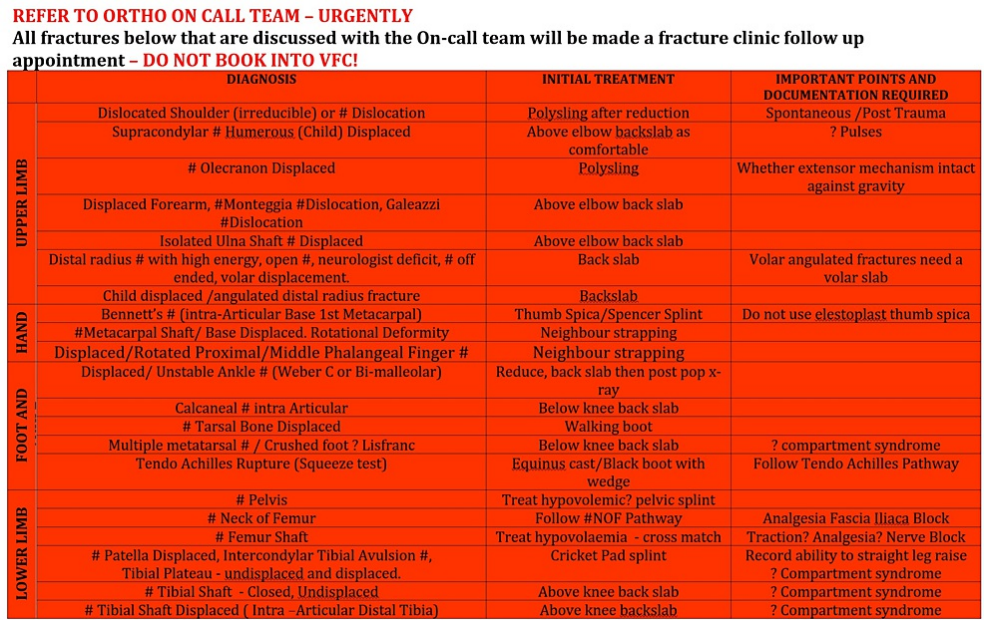
Virtual Fracture Clinic Emergency Fracture Management Matrix: Red Pathway Adapted from East Kent Hospitals University Foundation Trust Virtual Fracture Clinic Matrix for management of specific fractures, with permission. Designed by Jenni Finlay (Trauma Nurse Practitioner, Trauma and Orthopaedics Department, William Harvey Hospital, East Kent Hospitals University Foundation Trust).

Referrals for fractured femur, head injury, trauma calls, open fractures, complex wounds and the back pain pathway were excluded, as the T&O on-call team always reviews these patients under the red pathway of the VFC traffic light matrix (Figure 3).

The following data were collected for each referral that met the inclusion criteria: patient hospital number, diagnosis, referral source, reason for referral, plan, double booking with VFC and appropriateness, which was determined based on the VFC traffic light matrix (Figures 1, 2) and assessed by two reviewers independently. Appropriateness was defined as whether the pathway was followed for any given injury.

## Results

**Figure 4:**
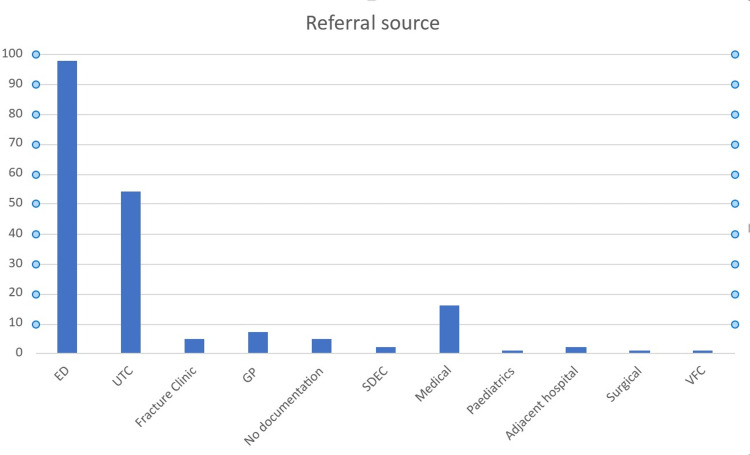
Sources of referrals ED: emergency department, UTC: urgent treatment centre, GP: general practice, SDEC: same-day emergency care, VFC: virtual fracture clinic

A total of 191 referrals to the Trauma and Orthopaedic Department were analysed. Most referrals were from the ED (51%) and UTC (23%) located within the hospital (Figure 4).

Of all patients referred who met the inclusion criteria, 27% went on to have surgical management. In addition, 30% were discharged with advice, and 35% were discharged with outpatient follow-up in a fracture clinic. Of the patients, 7% referred in line with the inclusion criteria were admitted. However, this does not reflect most T&O admissions at our institution, as patients with a fractured femur, head injury or admission via trauma calls were excluded.

**Figure 5:**
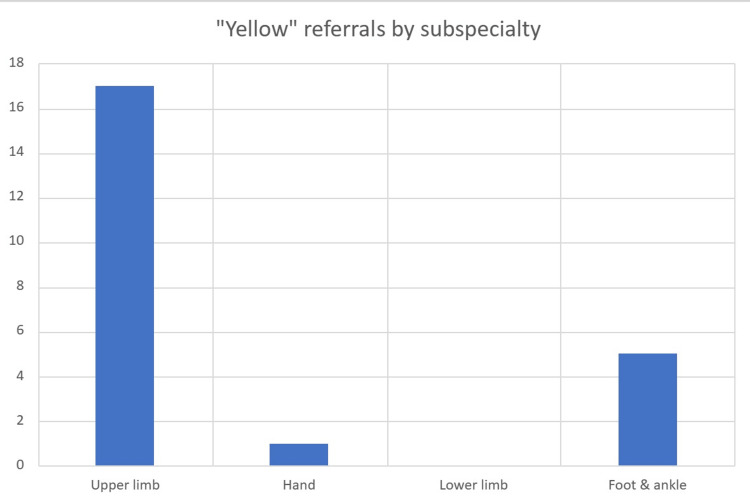
Referrals that should have been referred via the yellow arm of the VFC pathway divided by subspecialty VFC: virtual fracture clinic

Of the referrals, 39% were deemed to be inappropriate, and 35% of those deemed to be inappropriate referrals should have been referred to the VFC following the yellow arm of the pathway. Of those referrals, 50% were from the UTC, while 38% were from the ED. Of those, the majority were for upper limb referrals (Figure 5); 69% were from the UTC and 80% from the ED. Of the patients that were referred acutely who should have been on the green pathway, 86% were referred by the UTC, and 71% were sprains involving the lower limb. All patients referred that should have been on the green pathway did not have their radiographs reported at the time. The average time for a radiology report for those patients was seven days. No untoward events were identified. The average time to a report for the yellow box patients was two days with 29% being reported within 24 hours.

In addition, 7% were referred to the on-call team and VFC, duplicating review as these patients would have been discussed in the morning trauma meeting with the on-call consultant and reviewed by the consultant allocated to the VFC.

## Discussion

An established acute musculoskeletal pathway through MIU can reduce ED attendance, decrease wait times and ultimately improve patient satisfaction [13]. Despite a well-established VFC pathway, there were many referrals that could have been managed according to our Trust's protocol, which were referred to the T&O on-call team. Most referrals were within the yellow section of the traffic light system, with a smaller number in the green section (for discharge and written advice). The remaining referrals were deemed to be more appropriately managed by other services, such as general practice, emergency medicine, vascular, medical and surgical teams.

With uninitiated junior staff or trainees, referrals can often be to ask whether a patient should be referred to the virtual fracture clinic or to interpret radiographs and make a diagnosis when a report is unavailable. It is also important to remember that oftentimes, urgent treatment centres are staffed by emergency nurse practitioners (ENPs). This is not to say that nurse-led minor injuries units (MIUs) are inferior as there are multiple examples in the literature to suggest otherwise [14-16] but that there may be uncertainty in radiograph interpretation and hesitancy to discharge across both junior doctors and ENPs for fear of missing an important diagnosis.

It is known that errors in radiograph interpretation can occur in both the ED and UTC [17] and are more likely to occur in paediatric cases and the more junior the healthcare practitioner is. Evidence suggests that emergency nurse practitioners were at least as good as senior house officer (SHO)-level doctors at interpreting MIU skeletal radiographs [18,19]. There is evidence to suggest that junior doctors' accuracy in the interpretation of trauma radiographs is lower when compared to registrar or consultant counterparts [20,21]. Tachakra et al. (2002) [22] were able to demonstrate that emergency medicine consultants were at least as good as consultant radiologists at interpreting minor injury unit skeletal radiographs by contrast. Snaith et al. (2014) [23] found no significant discrepancy between emergency nurse practitioners and other medical staff for radiograph interpretation but noted a significant reduction in patient recalls and radiograph misinterpretation if radiographs are reported immediately by a reporting radiographer.

Two randomized controlled trials found ENPs to be at least equal to junior doctors and in some ways better in a minor injury unit setting including patient satisfaction, health education and clinical documentation [24,25]. It is reasonable to assume that some referrals, although managed via the VFC protocol, are referred for a second opinion when uncertainty exists or there is not yet a radiology report, to ensure optimal patient safety and the best patient outcome.

Concerning radiograph interpretation and discharges, Howard and Craib (2022) [26] looked at radiographer-led discharges once no fractures were identified. This strategy reduces waiting times and negates the need for an unsure practitioner to chase a radiology report or seek an orthopaedic opinion in a patient with a minor soft tissue injury. Instead, the patient would be discharged with appropriate advice following a pre-determined pathway.

There are also examples of advanced physiotherapist practitioner (APP)-led MIUs [27,28] that have done comparably well against emergency nurse practitioners when it comes to patient satisfaction and performance. The cost and clinical effectiveness of such strategies are yet to be determined within the literature, but for musculoskeletal complaints, the use of advanced physiotherapist practitioners (APPs) seems viable.

The other possible explanation for inappropriate referrals is admittedly an ambiguous referral matrix. The authors admit that the yellow box places the decision-making power in the hands of the ED doctor/UTC practitioner as it requires them to identify the injury as safe for discharge and follow-up in the VFC. A simplified system incorporating the yellow box into the green box may not mitigate this problem as the decision still falls onto the ED/UTC practitioner for those specific diagnoses. An alternative would be for the acute on-call team to take up the "yellow" cases, which are currently posing a challenge; however, this negates the benefits of the VFC pathway. The inclusion of Arbeitsgemeinschaft für Osteosynthesefragen/Orthopaedic Trauma Association (AO/OTA) classification in the matrix may help specify injuries but could also complicate the matrix and increase calls to the on-call team for clarification on which fracture subtype a particular injury is. While such classifications could be implemented into the matrix, this would require considerable educational efforts regarding various fracture classification subtypes directed toward the ED and UTC who do not typically use these systems.

Research has repeatedly shown that medical image interpretation among juniors is lacking and educational drives through e-learning and formal structured courses delivered by radiologists should be explored by all trainees as opposed to only on-the-job training [29]. Across the hierarchy of ENPs on the other hand, according to the Urgent and Emergency Care Emergency Practitioner Framework, all emergency practitioners including trainees would have been required to at least attend an interpretation of images course followed by ongoing continuing professional development (CPD) needs [30].

There are courses available aimed specifically at junior doctors and as refreshers for minor injury unit ENPs and GPs for radiograph interpretation. Based on our work, upper limb fractures need the most work, and CPD should be explored in this regard. Frequent, repetitive education, CPD and inter-departmental collaboration are the most viable options in the authors' opinion moving forward. Our department has initiated an education and awareness drive, redistributing posters of the VFC matrix and taking a personal non-confrontational approach to educating those unaware of it with the help of our trauma nurse practitioner. The matrix is always open to revision and improvement, and perhaps, more feedback from the practitioners who use it is required to point out areas that need clarification or are too complex. A website has also since been developed that is pending approval for trust use for ease of access to the VFC Matrix and to use as an educational e-learning tool. Immediate radiograph reporting may also be helpful and can reduce patient waiting times.

## Conclusions

No referral system is perfect, and expecting a complete absence of human error is unreasonable. However, ensuring an efficient and safe referral pathway means that overall patient care is improved. The authors believe that collaboration between UTC, T&O and ED with educational efforts and feedback can help broaden knowledge, improve awareness of our VFC pathway and improve patient care. This can be aided through the digitalization of the VFC Matrix for ease of access.
